# Antibacterial and Synergy of Berberines with Antibacterial Agents against Clinical Multi-Drug Resistant Isolates of Methicillin-Resistant *Staphylococcus aureus* (MRSA)

**DOI:** 10.3390/molecules170910322

**Published:** 2012-08-29

**Authors:** Guo-Ying Zuo, Yang Li, Jun Han, Gen-Chun Wang, Yun-Ling Zhang, Zhong-Qi Bian

**Affiliations:** 1 Research Center for Natural Medicines, Kunming General Hospital, PLA, Kunming 650032, China; Email: zuoguoying@263.net (G.-Y.Z.); liyang1227@126.co (Y.L.); kmwgc12@126.com (G.-C.W.); zhangyunling@126.com (Y.-L.Z.); 2 Kunming Medical College, Kunming 650032, China; 3 School of Basic Medical Sciences, Yunnan Traditional Chinese Medical College, Kunming 650500, China; Email: hanzjn@126.com; 4 Center for Infectious Diseases, Kunming General Hospital, PLA, Kunming 650032, China

**Keywords:** anti-MRSA, synergy, 8-acetonyl-dihydroberberine, levofloxacin, FICI

## Abstract

Antibacterial activity of berberine (Ber) and 8-acetonyl-dihydroberberine (A-Ber) alone and combined uses with antibacterial agents ampicillin (AMP), azithromycin (AZM), cefazolin (CFZ) and levofloxacin (LEV) was studied on 10 clinical isolates of SCCmec III type methicillin-resistant *Staphylococcus aureus* (MRSA). Susceptibility to each agent alone was tested using a broth microdilution method and the chequerboard and time-kill tests for the combined evaluations, respectively. The alone MICs/MBCs (μg/mL) ranges were 32–128/64–256 (Ber) and 32-128/128-512 (A-Ber). Significant synergies were observed for the Ber (A-Ber)/AZM and Ber (A-Ber)/LEV combinations against 90% of the tested MRSA strains, with fractional inhibitory concentration indices (FICIs) values ranged from 0.188 to 0.500. An additivity result was also observed for the Ber/AZM combination by time-kill curves. These results demonstrated for the first time that Ber and A-Ber enhanced the *in vitro* inhibitory efficacy of AZM and LEV to a same extent, which had potential for further investigation in combinatory therapeutic applications of patients infected with MRSA.

## 1. Introduction

The first clinical isolate of methicillin-resistant *Staphylococcus aureus* (MRSA) was reported in 1961 when only a year after methicillin was introduced for clinical use [1]. Presently the spread of MRSA (the so called “superbug” as it was originally termed) is of great concern in the treatment of staphylococcal infections, since it has quickly acquired resistance to all antibacterial agents, including even the emergence of glycopeptide resistant strains such as vancomycin-resistant *S. aureus* (VRSA) [2].

MRSA has become the most common cause of infections among many global pathogenic bacteria. Many life-threatening diseases could be attributed to MRSA, such as endocarditis, pneumonia, toxin shock syndrome. In our hospital, MRSA could be examined in over 80 percent sputum samples of pneumonia from severe and elderly patients in the intensive care unit (ICU). Therefore, the search for novel anti-MRSA agents with novel mode of action is urgently needed. Plants have evolved and accumulated an elaborately useful source of anti-infective drugs [3,4]. The therapeutic potential of phytochemicals has been increasingly recognized in the development of anti-MRSA agents [5]. In recent years, we have been working on searching for anti-MRSA compounds from the Chinese herbal medicines [6,7,8] and the interactions of the compounds with conventional antibacterial agents [9,10].

**Figure 1:**
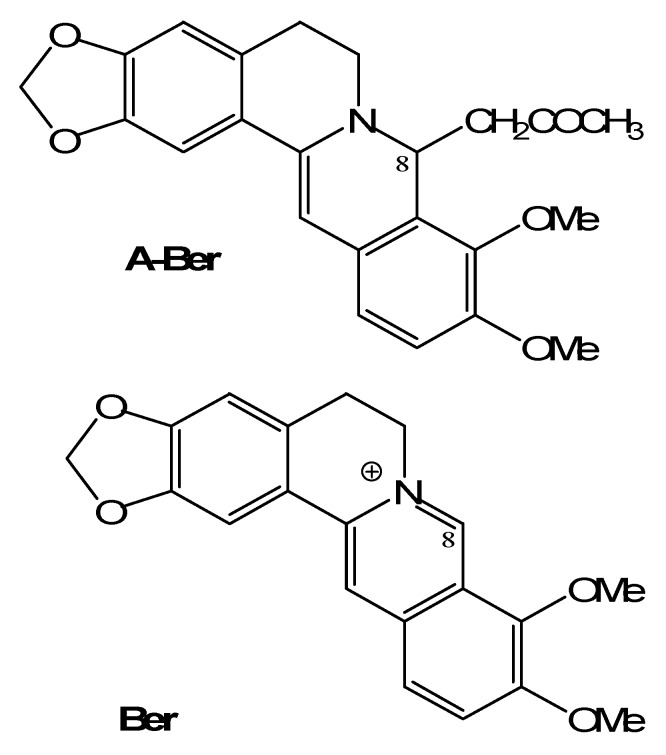
The structures of berberine (**Ber**) and 8-acetonyl-dihydroberberine (**A-Ber**).

Berberine is an isoquinoline alkaloid from many *Berberis* and *Mahonia* spp. (Berberidaceae) and other spp. in several different families, such as *Coptis chinensis* Franch (Ranunculaceae) and *Phellodendron amurense* Ruprecht (Rutaceae). It is a classic plant antimicrobial which has been used in the treatment of gastroenteritis, diarrhea, and cholera diseases [11]. However, its low solubility limits the bioavalability. The present report deals with the comparison of anti-MRSA activity between berberine (Ber) and its synthetic derivative 8-acetonyl-dihydroberberine (A-Ber) (Figure 1). Their synergistic effects on four conventional antibacterial agents ampicillin (AMP), azithromycin (AZM), cefazolin (CFZ) and levofloxacin (LEV) are also involved.

## 2. Results and Discussion

The *in vitro* Anti-MRSA activities of the two berberines (A-Ber and Ber) and four antibacterial agents representing four types of conventional antibacterial agents, *i.e.*, β-lactam (AMP), macrolide (AZM), cephem (CFZ) and fluoroquinolone (LEV) against 10 clinical MRSA isolates of SCCmec III type used alone are shown in Table 1. The MICs/MBCs (μg/mL) ranges were 32–128/128–512 and 32–128/64–256 for A-Ber and Ber alone and their (MICs)_90_ were 64 and 128 μg/mL, respectively. The agents’ order of potencies by (MICs)_90_ (μg/mL) followed LEV (32) > A-Ber (64) > Ber (128) = AMP (128) > CFZ (256) >> AZM (4,000). Compared with Ber, A-Ber possibly has higher membrane permeability under cell physiological conditions, so it showed better antibacterial activity against MRSA isolates [12].

**Figure 2:**
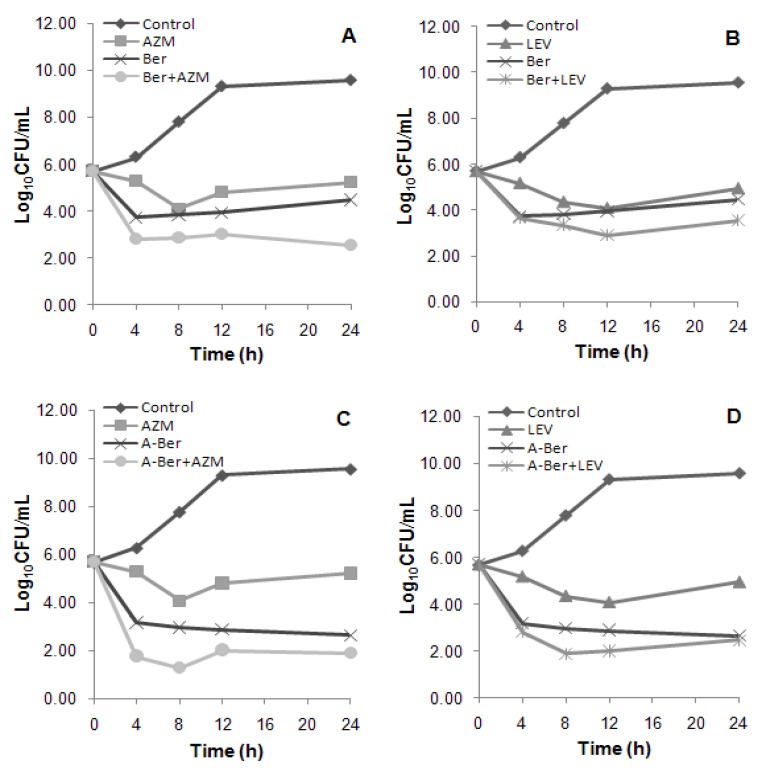
(**A**) The viable cells counts reduced 1.92; (**B**) The viable cells counts reduced 0.92; (**C**) The viable cells counts reduced 0.77; (**D**) The viable cells counts reduced 0.21. Time-kill curves of the synergistic effect of the combination at 1 × MIC (alone) concentration of berberine (Ber) and 8-acetonyl-dihydroberberine (A-Ber) with azithromycin (AZM) (**A** and **C**) and levofloxacin (LEV) (**B** and **D**), respectively against MRSA 004, a clinical MRSA strains of SCCmec III type.

Synergistic interactions of the berberines with the four antibacterial agents against the ten MRSA isolates were evaluated by the chequerboard method [11] and their fractional inhibitory concentration indices (FICIs) are listed in Table 2. Further results of LEV and AZM with the berberines through dynamic time-killing curves against MRSA 004 (one of the 10 isolates) are shown in Figure 2.

The chequerboard method showed significant synergies for the Ber (A-Ber)/AZM and Ber (A-Ber)/LEV combinations against 90% of the tested MRSA strains, with FICIs values ranged from 0.188 to 0.500. The MICs of berberines/antibacterial agents (AZM and LEV) combinations reduced by 50.0%–96.9% (Table 2). But the berberines/(AMP or CFZ) combinations all showed indifference (FICIs 1.5–2.0) (data not shown). The order of synergy effects followed the combinations (the lowest FICI) of A-Ber/AZM (0.156) > A-Ber/LEV (0.188) = Ber/AZM (0.188) > Ber/LEV (0.375) (Table 2). Therefore, the synergistic effects of A-Ber are generally higher than those of Ber when they were combined with the antibacterial agents. The phenomenon is also demonstrated by the other FICIs in Table 2. It might also be that the increased lipophilic property of A-Ber caused its increased synergy effect on AZM and LEV [12].

It is noted that the MICs of Ber alone are consistent with previously reported results, but the indifference effect of Ber/AMP combination in this study is different from the additivity in the literature [11]. This might be due to the different resistance profile of SCCmec III type MRSA strains tested in this study. The SCCmec III type MRSA is the major nosocomical isolate in Asian countries and characteristic for its multi-drug resistant not only to β-lactams but also to other types of antibacterial agents currently used [13].

In the time-kill analyses, synergistic effects of the combinations between the berberines and antibacterial agents were different from those found in the chequerboard method following the criterion of the synergy test (see Section3.5 in the experiment part). Time-kill curves (Figure 2) showed the Ber/AZM and A-Ber/AZM combinations resulted in an increase in killing of 1.92 (additivity) and 0.77 (indifference) log_10_CFU/mL of the colony counts at 24 h in comparison with that of the berberines (the most active) alone, while the Ber/LEV and A-Ber/LEV combinations resulted in much smaller increase of 0.92 and 0.21 (both indifference), respectively (Figure 2). Compared with the resulted killing of the antibacterial agents (the next most active) alone, the increased log_10_CFU/mL (combined) values followed the order of 3.34 (A-Ber/AZM) (C) > 2.68 (Ber/AZM) (A) > 2.49 (A-Ber/ LEV) (D) > 1.39 (Ber/LEV) (B) (Figure 2). Hence, bactericidal efficiency of the combinatory schemes were much more potent than those of the antibacterial agents alone, which is in agreement with the bacteriostatic results by chequerboard evaluation (Table 1 and Table 2). It has been confirmed that the overestimate of synergy experienced with the chequerboard test, and synergy testing performed by time-kill kinetics was used to confirm the results of chequerboard MIC testing [14].

**Table 1:** MICs and MBCs (g/mL) of Ber and A-Ber and four antibacterial agents alone against 10 clinical MRSA strains of SCCmec III type *^a^*.

Strain NO.		MRSA 004	MRSA 055	MRSA 123	MRSA 144	MRSA 189	MRSA 240	MRSA 276	MRSA 294	MRSA 328	MRSA 330	MRSAs (50%)	MRSAs (90%)	ATCC 25923
Ber *^b^*	MIC	64	128	32	128	64	64	32	32	32	64	64	128	64
	MBC	128	256	256	256	128	64	128	64	256	256	128	256	128
A-Ber	MIC	64	32	64	64	64	128	32	32	32	32	32	64	64
	MBC	512	256	256	512	128	128	128	128	256	256	256	512	512
AMP	MIC	64	128	64	64	64	64	64	128	128	64	64	128	16
	MBC	512	512	256	256	512	512	512	512	512	512	512	512	64
CFZ	MIC	128	128	128	256	256	128	256	128	128	256	128	256	128
	MBC	nt	nt	nt	nt	nt	nt	nt	nt	nt	nt	nt	nt	nt
LEV	MIC	8	16	32	16	32	16	16	16	16	8	16	32	2
	MBC	64	64	64	64	64	64	64	64	64	64	64	64	8
AZM	MIC	4,000	4,000	2,000	4,000	4,000	4,000	4,000	4,000	2,000	4,000	4,000	4,000	4,000
	MBC	Nt *^c^*	nt	nt	nt	nt	nt	nt	nt	nt	nt	nt	nt	nt
VAN	MIC	1	1	1	1	1	1	1	1	1	1	1	1	1
	MBC	2	2	2	2	2	2	2	2	2	2	2	2	2

*^a^* The tested maximum concentration of agents was 4,000 μg/mL. *^b^* Ber: berberine; A-Ber: 8-acetonyl-dihydroberberine; AMP: ampicillin; CFZ: cefazolin; LEV: levofloxacin; AZM: azithromycin. VAN: Vancomycin. *^c^* nt: not determined.

**Table 2:** MICs (g/mL) and FIC indices (FICIs) of berberines in combination with AZM and LEV against 10 clinical MRSA strains ofSCCmec III type.

Strain NO.	MRSA	MRSA	MRSA	MRSA	MRSA	MRSA	MRSA	MRSA	MRSA	MRSA	MRSAs	MRSAs
	004	055	123	144	189	240	276	294	328	330	(50%)	(90%)
Ber *^a^*	16	16	4	32	16	16	8	8	8	16	16	16
LEV	2	4	8	4	8	8	4	2	2	2	4	8
Effect	syn	syn	syn	syn	syn	add	syn	syn	syn	syn	syn	syn
FICI *^b^*	0.500	0.375	0.375	0.500	0.500	0.750	0.500	0.375	0.375	0.500	0.500	0.500
Rd% (Ber) *^c^*	75.0	87.5	87.5	75.0	75.0	75.0	75.0	75.0	75.0	75.0	>75.0	>75.0
Rd% (LEV)	75.0	75.0	75.0	75.0	75.0	50.0	75.0	87.5	87.5	75.0	>75.0	>75.0
Ber	16	16	4	16	8	32	8	8	4	8	8	16
AZM	500	500	250	1000	1000	500	500	1000	250	250	500	1000
FICI	0.375	0.250	0.250	0.375	0.375	0.625	0.375	0.500	0.250	0.188	0.375	0.500
Effect	syn	syn	syn	syn	syn	add	syn	syn	syn	syn	syn	syn
Rd% (Ber)	75.0	87.5	87.5	87.5	87.5	50.0	75.0	75.0	87.5	87.5	87.5	>75.0
Rd% (AZM)	87.5	87.5	87.5	75.0	75.0	87.5	87.5	75.0	87.5	93.8	>87.5	>75.0
A-Ber	8	4	4	16	8	32	8	8	4	8	8	16
LEV	2	2	4	4	2	4	4	4	4	2	4	4
FICI	0.375	0.250	0.188	0.500	0.188	0.500	0.500	0.500	0.375	0.500	0.375	0.500
Effect	syn	syn	syn	syn	syn	syn	syn	syn	syn	syn	syn	syn
Rd% (A-Ber)	87.5	87.5	93.8	75.0	87.5	75.0	75.0	75.0	87.5	75.0	>87.5	>75.0
Rd% (LEV)	75.0	87.5	87.5	75.0	93.8	75.0	75.0	75.0	75.0	75.0	>75.0	>75.0
A-Ber	16	4	16	8	16	32	8	8	8	8	8	16
AZM	125	125	250	1000	1000	1000	500	125	500	250	250	1000
FICI	0.281	0.156	0.375	0.375	0.500	0.500	0.375	0.281	0.500	0.313	0.375	0.500
Effect	syn	syn	syn	syn	syn	syn	syn	syn	syn	syn	syn	syn
Rd% (A-Ber)	75.0	87.5	75.0	87.5	75.0	75.0	75.0	75.0	75.0	75.0	>75.0	>75.0
Rd% (AZM)	96.9	96.9	87.5	75.0	75.0	75.0	87.5	96.9	75.0	93.8	>87.5	>75.0

*^a^* Ber: berberine; A-Ber: 8-acetonyl-dihydroberberine; AZM: azithromycin; LEV: levofloxacin. *^b^* FICI ≤ 0.5, synergy (syn); 0.5 < FICI ≤ 1, additivity (add); 1 < FICI ≤ 2, indifference (ind). *^c^* Rd%: % of MIC reduced = (MIC_alone_ − MIC_combined_) × 100/MIC_alone_.

This is the first report of anti-MRSA alone and antibacterial agent combinatory properties of A-Ber so far to the best of our knowledge [11]. The interactions of the berberines with different antibacterial agents might be attributed to the block of different resistant mechanisms of bacteria, including the bacterial efflux pump inhibitory effect of berberines [15,16]. As the clinical MRSA infections have become an increasingly pressing global problem, anti-MRSA synergistic effects between plant natural compounds and conventional antibacterial agents has further been demonstrated here as a promising way of overcoming current antibacterial agents resistance [17].

## 3. Experimental

### 3.1. Antibacterial Agents

Four antibacterial agents represented different conventional types were purchased from the manufacturers, *i.e.*; AMP (North China Pharmaceutical Co., Ltd, Shijiazhuang, China), CFZ (Harbin Pharmaceutical Co., Ltd, Harbin, China), AZM and LEV (Yangzhijiang Pharmaceutical Co., Ltd, Taizhou, China). Vancomycin (VAN) (Eli Lilly Japan K.K., Seishin Laboratories, Kobe, Japan) was used as the positive control agent. Cefoxitin disks were purchased from Tiantan biological products Co., Ltd (Beijing, China). A-Ber was synthesized from Ber (Changzhou Yabang Pharmaceutical Co., Ltd, Changzhou, China) following the procedure previously reported (data not shown) [12,18].

### 3.2. Bacterial Strains

MRSA strains (ten isolates with SCCmec III genotype) were obtained and characterized from the infectious sputum samples of critically ill patients in Kunming General Hospital [19,20,21]. The presence of mecA gene and SCCmec genotypes were determined by multiplex PCR methods at Kunming Institute of Virology, PLA, China, as previously reported [22]. ATCC 25923 was used as the control strain.

### 3.3. Media

Standard Mueller-Hinton agar and broth (MHA and MHB, Tianhe Microbial Agents Co., Hang Zhou, China) were used as bacterial culture media. MHB was used for all susceptibility and synergy experiments. Colony counts were determined using MHA plates.

### 3.4. Susceptibility Testing

MICs/MBCs were determined by standard broth microdilution techniques with starting inoculums of 5 × 10^5^ CFU/mL according to CLSI guidelines and incubated at 35 °C for 24h [7,23,24]. They were determined in duplicate, with concentrations ranging up to 4,000 μg/mL for AZM.

### 3.5. Synergy Testing

Potential anti-MRSA synergy was determined by FICI with chequerboard method and by time-killing analysis as previously reported [11]. The FIC of the combination was calculated through dividing the MIC of the berberines/antibacterial agents’ combination by the MIC of berberines or of the antibacterial agents alone, and the FICI was obtained by adding the FIC of berberines and that of antibacterial agents. The FICI results were interpreted as follows: FICI ≤ 0.5, synergy; 0.5 < FICI ≤ 1, additivity; and 1 < FICI ≤ 2, indifference (or no effect) and FICI > 2, antagonism [11]. In the killing curves, synergy was defined as ≥2 log_10_ CFU/mL increase in killing at 24 h with the combination, in comparison with the killing by the most active single drug. Additivity was defined as a 1–2 log_10_ CFU/mL increase in kill with the combination in comparison with the most active single agent. Indifference was defined as ±1 log_10_ CFU/mL killing or growth. Combinations that resulted in >1 log_10 _CFU/mL bacterial growth in comparison with the least active single agent were considered to represent antagonism [25,26]. All experiments were performed in triplicate.

## 4. Conclusions

In conclusion, this study demonstrated that Ber and A-Ber enhanced the *in vitro* inhibitory efficacy of AZM and LEV, which had potential for combinatory therapy of patients infected with MRSA and warrant further pharmacological investigation.
